# Electromagnetic Fields, Oxidative Stress, and Neurodegeneration

**DOI:** 10.1155/2012/683897

**Published:** 2012-09-09

**Authors:** Claudia Consales, Caterina Merla, Carmela Marino, Barbara Benassi

**Affiliations:** Unit of Radiation Biology and Human Health, ENEA-Casaccia, Rome 00123, Italy

## Abstract

Electromagnetic fields (EMFs) originating both from both natural and manmade sources permeate our environment. As people are continuously exposed to EMFs in everyday life, it is a matter of great debate whether they can be harmful to human health. On the basis of two decades of epidemiological studies, an increased risk for childhood leukemia associated with Extremely Low Frequency fields has been consistently assessed, inducing the International Agency for Research on Cancer to insert them in the 2B section of carcinogens in 2001. EMFs interaction with biological systems may cause oxidative stress under certain circumstances. Since free radicals are essential for brain physiological processes and pathological degeneration, research focusing on the possible influence of the EMFs-driven oxidative stress is still in progress, especially in the light of recent studies suggesting that EMFs may contribute to the etiology of neurodegenerative disorders. This review synthesizes the emerging evidences about this topic, highlighting the wide data uncertainty that still characterizes the EMFs effect on oxidative stress modulation, as both pro-oxidant and neuroprotective effects have been documented. Care should be taken to avoid methodological limitations and to determine the patho-physiological relevance of any alteration found in EMFs-exposed biological system.

## 1. Introduction

Over the past several decades people have been constantly exposed to electric (E) and magnetic (H) fields from both industrial and domestic uses. The EMFs are produced not only for technological applications (e.g., power lines mobile phones), but they are now widely used also in medicine for diagnostic (e.g., magnetic resonance imaging (MRI) scanner and microwave imaging) and therapeutic purposes (e.g., radiofrequency and microwave ablation and hyperthermia) [1, 2].

The increased social and public interest in this subject, based on the epidemiological data associating the extra risk of amyotrophic lateral sclerosis (ALS), childhood leukemia, adult brain cancer, and miscarriage with the EMFs exposure of the power line radiation [3–9], prompted the World Health Organization (WHO) Report (2007) and WHO Environmental Health Criteria (EHC) Report (2007) to issue precautions against the ELF-EMFs [10, 11].

### 1.1. EMFs Spectrum and Physical Interaction Quantities

**Figure 1:**
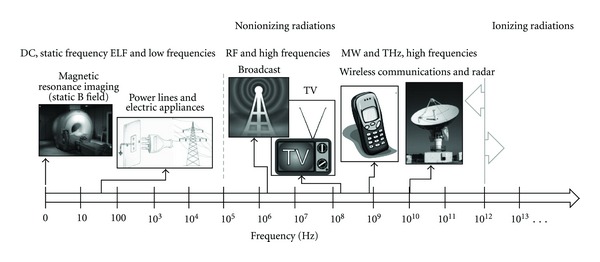
The whole electromagnetic spectrum, with partition between nonionizing and ionizing radiations, is reported. Main filed sources at the different frequencies are also sketched.

The EMFs coupling with biological systems depends on the frequency range of the employed signals, as well as on their characteristics as amplitude, modulation, waveform and polarization [12]. Mainly three categories of EMFs signals can be identified. They are classified as static, electric and magnetic fields (as direct current, DC, 0 Hz), Extremely Low Frequency fields (ELF, between 1 Hz up to 100 kHz) and high frequency (HF) fields, in the band of the Radio Frequency fields (RF, 100 kHz–3 GHz), and of the microwaves (MW, above 3 GHz) [13, 14]. These radiations (with frequencies below 300 GHz) are all nonionizing ones (Figure 1).

The established regulations against health hazards [13, 14] are based on two key mechanisms of interaction with biological systems, one elicited by DC and ELF sources, and the other by RF and MW exposures. For DC and ELF exposures, the induced E-field (V/m) and current density (J, A/m^2^) are the main physical quantities to describe the EMF interaction. They can be generated by both external applied E-fields and variable H-fields, and their amplitudes have to be limited in order to avoid hazardous health effects (e.g., magnetophosphenes induction, cardiac fibrillation, muscle and nerve contraction, and fulguration) [12]. When RF and MW exposures are taken into account, the main mechanism to be considered is the rise in temperature, as no charges movements are triggered at these frequencies. The heat effect is strictly dependent on both the water content of the biological target, the frequency, and intensity of the electromagnetic (EM) radiation. Therefore, for RF and MW exposure, the characteristic interaction quantity is the Specific Absorption Rate (SAR) [12], defined as the power (W) deposited by an EM radiation in a unitary mass (g) of the biological target, in a fixed time period(s), and it is measured in Wkg^−1^.

### 1.2. Interaction of the EMFs with the Biological Systems

As EMFs are nonionizing, the search for conventional genotoxic mechanisms, as potentially responsible events underlying the interaction with the biological systems, have shown contradictory results. A convincing molecular mechanism, disclosing the link between human diseases and exposure to electromagnetic fields, is still lacking, although change in cell cycle, induction of cell death, modification of protein expression, and mainly oxidative stress have been proposed [15–18]. 

Metabolic processes which generate oxidants and antioxidants can be influenced by environmental factors, such as EMFs [19]. Increased EMFs exposure can modify the cellular balance by generating reactive oxygen species (ROS) [20–24]. Physical processes at atomic level are indeed the basis of reactions between biomolecules and EMFs, as the field can magnetically affect chemical bonds between adjacent atoms and alter the energy levels and spin orientation of electrons. Overproduction of ROS can damage cellular components, mainly lipids in membranes and nucleic acids. Moreover, ROS can harm cells by depleting enzymatic and/or nonenzymatic antioxidants triggering progressive dysfunction and eventually genotoxic events [25–27].

This redox-related mechanism has been mainly documented for the ELF-EMFs. Scaiano et al. [23] first proposed that ELF-EMFs exposure can stabilize free radicals in such a way as to increase their lifetime and permit a wider dispersion rather than their return to the basal level. This might contribute to an increase in the activity and concentration of the free radicals, as also reported in the immune system, mainly mouse macrophages, human monocytes, and rat neutrophils [28–31]. Simkó et al. [31] in particular, demonstrated an increased phagocytic activity and an enhanced super oxide production in mouse macrophages after ELF exposure, in a dose-dependent manner. Besides, the inhibitory potential of chronic ELF-EMFs exposure on the availability of the pineal gland hormone melatonin, which physiologically acts as a radical scavenger, has been suggested as an additional pathway in the oxidative stress-driven interaction of ELF with the biological systems [32, 33]. ELF-EMFs might be therefore a stimulus to induce an “activated state” of the cells, such as in the phagocytic activity, which enhances the release of free radicals, and can eventually turn into a genotoxic event following chronic exposure. The suppression of the ELF-enhanced cell proliferation in the presence of radicals scavengers, as shown by Katsir and Parola in chick embryo fibroblasts [34], represents an another supportive finding for this proposed model of interaction between EMFs and biological systems via ROS generation.

The biological response induced by HF-EMFs, mainly RF exposure, may be instead explained by two distinct interaction mechanisms: thermal effects (that rely on the ability of RF fields to transfer their energy to biological matter, leading to an increase in average temperature through the vibration of atoms and molecules) and nonthermal effects [35, 36]. The latter only have been correlated to the generation of oxidative stress. 

Nonthermal effects range from alterations in the permeability of the blood-brain barrier, to changes in encephalogram and blood pressure, although the matter is still controversial [37, 38]. The greatest mystery about these nonthermal effects is their lack of a theoretical basis, and, from an experimental point of view, a major problem in their definition is how to distinguish them from direct and indirect thermal effects. Oxidative stress has been proposed as the underlying mechanism responsible for this kind of RF effects, although the results are still controversial. In this context, it has been proposed that RF-EMFs (875 MHz, 0.07 mW/cm^2^) generate extracellular ROS by stimulating cell membrane nicotinamide adenine dinucleotide (NADH) oxidase in Rat1 and HeLa cells *in vitro* [15]. ROS then activate metalloproteases on the outer surface of the cell, which cleave membrane-anchored progrowth factors and trigger the activation of p38 as well as the ERK (extracellular-signal-regulated kinase) mitogen-activated protein kinases (MAPKs) [15]. An enhanced production of ROS after combined exposure to RF radiation (930 MHz, SAR 1.5 Wkg^−1^) and iron ions was also reported in an experimental model of rat lymphocytes [39], and induced lipid peroxidation, accompanied by decreased activity of superoxide dismutase (SOD), myeloperoxidase (MPO) and glutathione peroxidase (GSH-Px) by RF exposure has been reported in various organs, such as rat kidney and guinea pigs liver [18, 40]. Moreover, in the latter animal model, treatment with epigallocatechin-gallate, the main active component of green tea, and N-acetyl cysteine, a glutathione (GSH) precursor, provided protection against oxidative stress-induced liver injury caused by RF-EMFs [40].

However, it should be noted that no significant ROS generation was measured in other human cell lines when exposed to 1800 MHz (0.5–2 Wkg^−1^, for 30–45 min) [41, 42], and no short term activation of ERKs was detected in auditory hair cells treated for 15 min with RF-EMFs (1763 MHz, SAR 20 Wkg^−1^) [43]. Hence, both the generality of activation of classical MAPKs cascade by RF-EMFs and the validity of the proposed ROS-mediated mechanism are still challenged. Differences in cell lines and experimental methods, used for both *in vitro* and *in vivo* exposure, might explain, in part, these still conflicting findings.

## 2. EMFs and Oxidative Stress in Brain 

Free radicals are essential for physiological processes, especially in brain metabolism [44]. The brain consumes the highest amount of oxygen in the human body and, although most oxygen is converted into CO_2_ and water, a small amount of O_2_ forms ROS [45]. The high metabolic rate and the composition rich in polyunsaturated fatty acids which are ROS targets in brain, make this organ more sensitive to oxidative damage [46]. 

Here we aim at critically reviewing the scientific literature focused on the cross-talk between redox-driven biological systems and EMFs in brain and its pathologic degeneration.

### 2.1. Criteria for Reference Selection

This paper is an overview of the results arising from both the *in vitro* and *in vivo* studies that investigated whether the EMFs (both ELF and HF) exposure could affect the oxidative balance of cells in the central nervous system. The interest about this topic stems from the knowledge that oxidative stress is a hallmark of neurodegenerative diseases and the hypothetic influence of EMFs on the onset and/or progression of these pathologies is frequently debated. 

The search was carried out by consulting both PubMed data base and the official reports concerning the biological effects of the EMFs at the following websites:

 
http://efhran.polimi.it/docs/IMS-EFHRAN_09072010.pdf
 
http://ihcp.jrc.ec.europa.eu/our_activities/public-health/exposure_health_impact_met/emf-net/docs/reports/EMF%20NET%202.2_%20D4bis._pdf
 
http://ec.europa.eu/health/ph_risk/committees/04_scenihr/docs/scenihr_o_007.pdf
 
http://www.hpa.org.uk/webw/HPAweb&HPAwebStandard/HPAweb_C/1317133826368.

The PubMed search was conducted using combinations of the following search terms: (oxidative stress), (oxidative stress AND brain), (oxidative stress AND neurodegenerative disease) with (EMFs or ELF-EMFs or HF-EMFs). Publications about pulsed and/or static fields have not been considered. A new Pubmed search was then conducted for all authors previously identified, and the reference list of any additional papers examined. Papers have been classified considering the frequency of electromagnetic field analyzed, irrespective of the experimental models and conditions employed.

The whole search was last updated in May 2012.

All papers matching the above-mentioned criteria have been quoted and referenced throughout the paper, without assessing on the quality of methodology, even if a critical revision of the exposure methods and experimental conditions has been carried out in Section 4 of the present paper.

### 2.2. ELF-EMFs and Brain Oxidative Stress

The interaction between the ELF-EMFs and the biological systems directly implies the involvement of the oxidative stress, in particular by the radical pair mechanism, as the equilibrium of the elementary reaction producing a pair of radicals may be altered by the magnetic field [23, 47, 48]. Thus, ELF-EMFs may prolong the lifetime of free radicals and increase their concentration in living cells [20–27]. Although radical pair recombination has been well documented for different biological processes (such as several enzymatic activities or orientation ability of migratory birds) in response to environmental EMFs [49, 50], its role as candidate mechanism, underlying ELF ability to affect brain oxidative stress and disease, has not been detailed so far. 

ELF-EMFs exposure (50 Hz, 0.1–1.0 mT) is reported to elicit redox and trophic response in rat cortical neurons [51], and to induce oxidative stress in mouse cerebellum [52] (Table 1). In accordance, ELF-EMFs increase free radicals content with consequent lipid oxidative damage in brains of mice and rats [53, 54]. A contributing factor to the ELF-EMF-induced oxidative stress may be zinc deficiency, as lipid peroxidation-induced in Sprague-Dawley rats by long term exposure to ELF-EMFs (50 Hz, 50 mG) can be ameliorated through systemic antioxidant zinc supplementation [55]. 

Oxidative stress further arises from a disequilibrium between the production of free radicals and the scavenging capacity driven by various antioxidant compounds and enzymes, including catalase (CAT), glutathione (GSH), GSH-Px, and critically important in brain SOD [56]. All these antioxidant defense systems can be specifically deteriorated by the ELF-EMFs (60 Hz, 12 G, 3 hours), thus amplifying oxidative stress [56]. In particular, in an experimental model of rat brain, 50 Hz (100 and 500 *μ*T) exposure was reported to induce a severe toxic effect by impairing the catalase (CAT) antioxidant defense [57]. Also in combination to movement restriction, the chronic exposure to ELF-EMFs (60 Hz, 2.4 mT) was able to elicit both the impairment of CAT activity and a severe lipid peroxidation in brains of Wistar rats [58].

As an overall oxidative stress-based decline in physiologic functions and in resistance to stressors is an unavoidable consequence of aging [59], it has been also investigated whether the aging process per semight reduce resistance towards EMFs prooxidant attack. In this context, ELF-EMFs exposure (50 Hz, 0.1–1.0 mT) was shown to significantly affect antioxidant enzymatic capacity in both young and aged rat brains [60], with aged rats exhibiting a remarkable fall of all the major antioxidative enzymatic activities, thus pointing to a greater age-dependent susceptibility to EMFs-dependent oxidative stress. 

In this ELF-ROS-brain context, only one paper, to our knowledge, reported no effect following exposure of mice to ELF-EMFs (60 Hz, 0.2–1.2 mT) [61]. Kabuto et al. indeed demonstrated that no ROS generation nor lipid peroxidation could be detected in brain homogenates of exposed mice. Interestingly, they observed a slight decrease in oxidative damage in mice exposed to static field (2–4 mT).

### 2.3. HF-EMFs and Brain Oxidative Stress

Exposure to RF radiation (mainly from mobile phones) has been postulated to trigger a variety of neurological effects, including headaches, changes in sleep pattern, modification in the neuronal electrical activity, and disturbance in the neurotransmitter release [62]. Although still controversial, increasing evidence indicates that oxidative stress may be involved in the adverse effects elicited by RF-EMFs in the nervous system (Table 1). 

In favor of this hypothesis, Ilhan et al. [63] reported a marked oxidative damage in brain tissues of rats exposed to 900 MHz signal for GSM (Global System for Mobile communications) (SAR of 2 Wkg^−1^ in the brain) for 7 days. They first proved that RF-EMFs exposure of the brain in rats cause histopathological changes typical of brain injury, accompanied by oxidative stress, as biochemically revealed by increased levels of nitric oxide (NO), malondialdehyde (MDA), as well as xantine oxidase (XO), and adenosine deaminase (ADA) activities. Moreover, treatment with the antioxidant *Ginkgo biloba* extract, a potent free radical scavenger agent, significantly prevented oxidative damage and pathological alterations in brain tissues. 

In a different experimental model of guinea pigs, Meral et al. [64] evaluated the effects of GSM signal (890–915 MHz EMF, SAR 0.95 Wkg^−1^, for 12 h/day for 30 days) on the oxidative stress pathway, by assessing MDA, GSH, CAT and vitamin A, D_3_, and E (considered part of antioxidant defense systems of tissues) levels in both brain and blood. Authors reported an increase of MDA, and a decrease of both GSH and CAT levels in brains, without any modulation in vitamins concentration, thus suggesting that RF exposure could trigger depression of the antioxidant systems, due to increased lipid peroxidation and formation of free radicals. 

Also in a model of rats brain, locally exposed to GSM-900 MHz signal by a head loop antenna (SAR of 1.5 WKg^−1^ and 6 WKg^−1^), the activity of the cytochrome oxidase, a specific redox-sensitive enzyme and marker of neuronal functional activity in brain, was found compromised, but only at the higher SAR used, and exclusively in specific brain areas, such as frontal cortex, posterior cortex, hippocampus, and septum [65]. 

In the context of the *in vitro* studies, Xu et al. [66] exposed primary cortical neuronal cultures to a 1800 MHz field (SAR of 2 Wkg^−1^) for 24 hrs. They reported a significant increase of ROS production, and demonstrated, for the first time, a reduction in the mitochondrial DNA copy numbers. Interestingly, these effects could be reverted by pretreating cultures with melatonin, a pineal neurohormone with known antioxidant capacity. 

In contrast to these findings are the *in vivo *data reported by Irmak et al. [37]. They analyzed MDA, NO, ADA, XO, MPO, SOD, CAT, and GSH-Px levels in both brain and sera of RF-EMFs-exposed rabbits (900 MHz GSM signal, 2 W peak power, average power density 0.02 mWcm^−2^, for 30 min/day). Although an elevated activity of SOD and a reduction of NO levels were observed in the sera of exposed animals, no change in any brain parameters of rabbits was reported. In accordance, exposure of the dopaminergic neuroblastoma cell line (SH-SY5Y) to GSM (SAR of 5 WKg^−1^ for 1 hr) triggered no effects on GSH levels, nor induced DNA fragmentation, even if a significant increase in lipid peroxidation was observed [67]. 

## 3. EMFs and Neurodegenerative Diseases

Physiological dysfunction by oxidative stress leads to pathogenic condition. It is well established that free radicals can interact with DNA, leading to mutation, and interfere with gene regulation to eventually promote carcinogenesis [68]. But an additional aspect of free radicals is their potentiality to affect neuropathological conditions such as Parkinson's disease (PD) and Alzheimer's disease (AD), the oxidative stress being a molecular hallmark of neurodegenerative diseases [69].

Despite the increasing interest in this field of research and the epidemiological data suggesting the potential association between EMFs and neurodegeneration, the experimental findings supporting this link are still controversial, and dependent on both the field frequency applied and the disease investigated, as here reviewed.

### 3.1. EMFs Exposure and AD

 AD is the most common neurodegenerative disease, and is characterized by progressive loss of neurons, particularly in the cortex and hippocampus [70]. Oxidative damage has been implicated as a key mediator in the onset, progression and pathogenesis of AD. In particular, redox reactive metals, such as iron, are leading causes of redox-generated hydroxyl radicals, and can promote the synthesis of amyloid beta (A*β*) precursor protein in an oxidative stress-mediated pathway [27, 71, 72].

Despite the knowledge of AD molecular basis, the etiology of Alzheimer's is poorly understood. Many environmental and lifestyle factors, together with age, family history of dementia, and apolipoprotein E *ε*4 genotype have been hypothesized to increase the risk of developing AD [73]. Among the potential environmental factors, exposures to aluminium, solvents, pesticides, and lead and also EMFs (mainly ELF-EMFs) have been the most widely studied [74]. Several available epidemiological studies and meta-analysis data seem to suggest a potential association between occupational exposure to ELF-EMFs (typical of electric power installers and repairers, power plant operators, electricians, electric and electronical equipments repairer, telephone line technicians, welders, carpenters, and machinists) and AD onset [75–77], although their biological nexus remain unknown. Only suppositions have been proposed, involving melatonin and biosynthetic enzymes in the pineal gland, Ca^2+^ efflux in immune system cells and neurons, interference with the amyloidogenic process, and clearly oxidative stress [78–80]. Sobel and Davanipour [81] hypothesized that ELF-EMFs exposure might increase A*β* peripheral and brain production by modulating the Ca^2+^ channels. The proposed mechanism relied on the ability of the EMFs to increase the intracellular ion concentration levels, a molecular factor that positively correlates with the cleavage of the amyloid precursor protein to give the soluble A*β*. ELF would hence favor the production of A*β* secreted in the bloodstream.

A completely different scenario in the Alzheimer's response to EMFs has been recently proposed by Arendash et al. [82] (see Table 2). They first reported that long-term (7–9 months) RF-EMFs exposure, directly associated with cell phone use (918 MHz; 0.25 WKg^−1^), provide cognitive benefits, disclosing a potential noninvasive, nonpharmacological therapeutic strategy against AD. Several earlier studies have already evaluated the EMFs exposure at cell phone frequencies (900 MHz) in normal rodents, showing no effects on cognitive performance, but the exposure involved a short-term period (7–14 days) [83]. In Arendash' paper, both cognitive-protective and cognitive-enhancing effects, associated to reduced brain A*β* deposition and increased cerebral blood flow, were demonstrated in transgenic mice destined to develop AD over a long term exposure period, without increasing indices of oxidative stress in the brain. 

Arendash and colleagues recently extended their earlier findings by evaluating the impact of long term RF-EMFs treatment given to very old (21–26 month old) APPsw (amyloid precursor protein) and APPsw + PS1 (presenilin) mice, both bearing much heavier brain A*β* levels than the same animals used in their first publication. In these aged mice, with advanced A*β* pathology, long term RF exposure further revealed a profound ability to reverse brain A*β* deposition, to induce changes in the regional cerebral blood flow, and to provide selected cognitive benefits, all without induction of brain hyperthermia and without increase in brain oxidative stress [84]. 

It is worth noting that data from the same group attributed the long term-RF-dependent cognitive benefits to the enhancement of brain mitochondrial function of AD transgenic (Tg) animals [85]. They indeed reported that RF-EMFs treatment is able to reduce mitochondrial ROS generation and to enhance mitochondrial membrane potential in both cerebral cortex and hippocampus, but not in the striatum or amygdale, selectively in AD Tg mice. These findings are in contrast with what is stated in the other two publications (where they reported no change in the indices of brain oxidative stress), and leaves open the question whether RF benefits in AD involve oxidative stress.

In accordance to a potentially neuroprotective function elicited by RF, Söderqvist et al. [86] reported increased serum concentrations of transthyretin (TTR), a molecule specifically sequestering A*β* peptide, among long term users of wireless phone, in both a cross-sectional study of 313 subjects using mobile phones and cordless phone, and in a provocation study on 41 people exposed for 30 min to 890-MHz GSM signal (1.0 WKg^−1^), suggesting that TTR might be involved in the RF-mediated benefits in AD mice.

Further studies are needed to corroborate these findings, to elucidate the biological mechanism and to validate the therapeutic use of RF fields, if any. It must be pointed out that several other studies indicated an increased risk brain tumors in people with long-term use (≥10 years) of mobile phones, taking into account which side of the head the handset has been mostly used [92], thus highlighting how this issue is still controversial and requiring further investigations.

### 3.2. EMFs Exposure and PD

PD is the second most common neurodegenerative disease, relying on the loss of dopaminergic neurons in the substantia nigra in association with the occurrence of intracytoplasmic neuronal inclusions (Lewy bodies) of *α*-synuclein [93]. Oxidative stress, generated by dopamine redox chemistry and by *α*-synuclein mutation, is considered one of the pathogenic factors in PD [93]. The oxidative damage to lipids, protein, DNA, and elevated RNA oxidation have been observed in both postmortem substantia nigra tissue and cerebrospinal fluid from living PD patients [27]. 

Differently from AD epidemiology, there are poor epidemiological bases supporting an univocal association between PD and exposure to EMFs. A pilot study by Wechsler et al. [94] first suggested that PD may be induced by occupational exposure to EMF, although a too small number of subjects was included in the study. Subsequently, two retrospective cohort studies [95, 96] and a death certificate-based case-referent study [97] failed to find a convincing correlation between Parkinson's disease and occupational magnetic field exposure. The death certificate-based method only found modest risks for power plant operators and telephone installers and repairers [97]. In a study by Noonan et al. [98], welders, who are exposed to high levels of magnetic fields as well as to other potentially neurotoxic agents such as metals, accounted for some of the observed risk of PD, suggesting an association between welding and an increased risk to develop Parkinson's. Finally, a recent paper from Huss et al. [99], based on a cohort of 4.7 million people of the Swiss National Cohort, followed over the period 2000–2005, demonstrated no consistent association between mortality from Parkinson's disease and exposure to ELFs power lines (220–380 kV, 50 Hz). Therefore, up to date, convincing epidemiological data supporting a correlation between PD and environmental/occupational EMFs exposure are still lacking.

Given the contradiction in epidemiological studies, *in vitro* and *in vivo* experimental findings disclosing the potential PD-EMFs correlation, are very sparse. To our knowledge, only a recently released paper attempted to investigate whether oxidative stress might be triggered by EMFs exposure and thus affect PD etiology and/or progression [87] (Table 2). Authors used a highly (80%) neuron-enriched mixed cortical cell culture from brains of rat embryos to study the impact of chronic (on the scale of the *in vitro* studies) exposure to GSM-900 MHz, at a low SAR (0.25 WKg^−1^) [87]. Despite previous records, no ROS generation or oxidative damage were observed in the neuron-enriched experimental model following RF exposure, although authors reported the first evidence of an EMFs-mediated downregulation of the *α*-synuclein, probably by promotion of its deubiquitination [87].

### 3.3. EMFs Exposure and Amyotrophic Lateral Sclerosis

Amyotrophic Lateral Sclerosis is a fatal neurodegenerative disorder characterized by progressive degeneration of motor neurons in the spinal cord, motor cortex, and brainstem. About 5–10% of ALS display familial inheritance, but in the majority of patients there is no inherited link. Both familial (fALS) and sporadic ALS (sALS) produce similar pathological symptoms [100]. At molecular level, a mutation in the gene encoding the antioxidant Cu^2+^/Zn^2+^ SOD (SOD1) has been reported in about 20% of fALS patients [101], still indicating the key role exerted by the oxidative stress in this neuropathological disorder [102]. In accordance, mitochondrial dysfunction may play a more significant role in the etiopathogenesis of this disorder than previously thought. The complex physiology of mitochondria and the alteration of their properties might confer an intrinsic susceptibility to long-lived, postmitotic motor neurons to energy deficit, calcium mishandling, and oxidative stress [103].

Although several hypotheses concerning the pathogenesis of the ALS have been generated, the etiology of the vast majority of cases is unknown. Electrical exposure has been cited as a possible environmental risk factor. Haynal and Regli were the first to raise the hypothesis that exposure to ELF-EMFs was linked to ALS in 1964 [6]. Since then, other epidemiological studies have positively correlated ALS death with occupational exposure to EMFs (electric utility workers), with relative risks ranging from 2 to 5, while only a few studies found little or no association [5, 95–97, 104–106]. A recent UK study found no risk increases in any job categories for motor neuron disease mortality among electricity generation and transmission workers compared to the general population [107]. Also Parlett et al. [108] did not provide any evidence for an association between magnetic field exposure and ALS mortality. After adjusting for age, sex, and education, they reported no increased risks of ALS mortality in relation to potential magnetic field exposure.

Thus, the evidence linking electrical occupations to an increased risk in ALS is remarkably consistent, but the evidence of an association with measured magnetic field levels is weaker. Lack of assessment of magnetic field exposure at the workplace and possible confounding by electric shocks, were the major limitations. Therefore, pending further well-designed epidemiological studies, there is still a need for confirmation of the correlation EMFs exposure-ALS from specifically designed laboratory experiments.

To our knowledge, the paper from De Gannes et al. [88] (see Table 2) is the only experimental study carried out in an animal model, in a controlled magnetic environment. Mutated SOD-1 mouse experimental model (Tg-SOD1^G93A^), which is currently the most accurate animal model for studying ALS, was employed to assess the possible effects of chronic exposure to ELF-EMFs (2 hours/day, 5 days/week for 7 weeks, to 50 Hz, at 100 and 1000 *μ*T) on the development of this neurodegenerative disease. The exposure levels were chosen on the basis of the European recommendation setting limits of 100 *μ*T for public exposure and 500 *μ*T for workplace [88]. By monitoring body weight, motor function, and life span of mice over the exposure period, authors did not reveal any difference between exposed and control animals, providing no evidence of a link between ELF exposure and ALS in this oxidative stress-prone experimental model. Despite it being reported that the yield and nature of oxygen reactive species may be affected at magnetic field strength above 100 *μ*T, the reported lack of biological effect may reflect the fact the pathophysiology of the familial form, characterized by SOD-1 mutation, is probably different form the sporadic one, and does not proceed via oxidative stress at the dose/time chosen for the exposure. Whether longer exposures or exposure of younger animals would affect the outcome is unknown and requires further investigation.

### 3.4. EMFs and Huntington's Disease (HD)

Huntington's disease is an autosomal dominant, progressive neurodegenerative disorder characterized by an array of different psychiatric manifestations, cognitive decline, and choreiform movements. The underlying molecular genetic defect is an expanded trinucleotide (CAG)_n_ repeat encoding a polyglutamine stretch in the N-terminus of the huntingtin protein. In most cases, HD is fully penetrant. Although huntingtin is ubiquitously expressed, the mutated gene leads to selective neuronal cell death in the striatum and cortex, even though the mechanisms by which it triggers neuronal dysfunction and degeneration are not fully understood. Impaired ubiquitin-proteasome activity, defective autophagy-lysosomal function, transcriptional dysregulation, apoptosis, mitochondrial, and metabolic dysfunction have been shown to play important roles in the pathogenesis of HD, as well as oxidative stress, like in other neuropathologies [91, 109, 110]. 

The potential correlation between EMFs exposure and HD pathogenesis is not sustained by epidemiological evidence. A few papers from a single research group attempted to disclose their connection in a mouse model of HD pathogenesis achieved by administrating animals with the 3-nitropropionic acid (3NP). This toxin is a selective inhibitor of succinate dehydrogenase (SDH) in the complex II of the mitochondrial electron transport chain [111]. 3NP triggers energy impairment, cytotoxicity, oxidative stress, and, eventually, neuronal death. In addition, animals exhibit motor and cognitive changes similar to HD [112, 113]. Stimulation of rats with ELF-EMFs (60 Hz and 0.7 mT, 2 hours in the morning and 2 hours in the afternoon, for 8 days), given either before or after the 3NP administration, partially prevented or reversed the neurotoxin-induced oxidative stress. Besides, a reduction in cellular loss and an increase in SDH activity was also observed [89, 90] (see Table 2).

Further evidences by Tasset et al. [91] strengthened the hypothesis of a neuroprotective effect elicited by ELF-EMFs. In a rat model of 3NP-induced HD, behavior patterns as well as changes in neurotrophic factor, cell damage, and oxidative stress biomarker levels were monitored. Rats were given 3NP over four consecutive days (20 mg/kg body weight), whereas ELF-EMFs (60 Hz and 0.7 mT) were applied over 21 days, starting after the last injection of 3NP. If compared to control 3NP-treated animals, ELF-EMFs improved neurological scores, enhanced neurotrophic factor levels, and reduced both oxidative damage and neuronal loss. Moreover, exposure to electromagnetic fields alleviated 3NP-induced brain injury and prevented loss of neurons in rat striatum, thus showing considerable potential as a therapeutic tool.

Taken as a whole, these data support the hypothesis that magnetic stimulation in rats prompts an increase in neuron survival and/or in neuronal density; this would eventually lead to normalized functioning of the nervous system, evident in the recovery of behavior patterns similar to those of a healthy rat.

## 4. Comments and Perspectives

So far there is still no general agreement on the exact biological effect elicited by EMFs, on the physical mechanisms that may be behind their interaction with biological systems, or on the extent to which these effects may be harmful to humans. In particular ELF-EMFs, such as those generated by power lines, have been suggested to increase the risk of several human diseases, mainly neoplastic malignancies [7, 8, 114]. The International Agency for Research on Cancer (IARC) inserted ELF in the 2B section of the table of carcinogens (“possible") in 2001, and recently classified also the Radio Frequency (RF) fields as 2B [4, 115]. In addition, early studies seemed to indicate that ELF-EMFs could contribute to the etiology of neurodegenerative disorders, in particular of AD and ALS [6, 9, 74]. Hypotheses relating the EMFs to the neurodegenerative diseases are a relatively novel part of the EMF research area and, so far, only a modest number of studies have been performed if compared to cancer research field. 

However, this area has quickly acquired attention because of implications in human health, occupational exposure, and aging, although, for a number of methodological reasons, the epidemiology of neurodegenerative diseases is more difficult to study than cancer. The most obvious difficulty is that neurological diseases are not recorded in registries in the same way as cancers, and that the mortality registries are less reliable as sources of cases. There are also lack of consensus on diagnostic criteria and difficulties in assessing time of disease onset. In addition, there is also a gender implication in epidemiological studies on neurodegeneration. Women display the higher incidence in pathologies such as the AD, but it is hard to base a study on their occupational exposure, as women have less often been employed especially in those work categories where the exposition to EMFs is high. Moreover, in occupational studies, distinguishing between exposure to EMFs and to chemical agents is often problematical, as workers are frequently exposed to a combination of both of these potentially neurotoxic factors. A notable weakness in neurodegenerative disease studies is case identification. In some studies, cases were identified in hospitals and controls among patients with other diseases in the same hospitals or among friends or relatives of cases. These studies are likely to have greater potential for selection bias than population-based studies, which, on the other hand, have often identified cases from mortality registries and thus have greater potential for disease misclassification. These and other difficulties are reflected in the literature, and the studies that have best avoided these limitations often suffer from small number.

Moreover, another important issue in the epidemiological studies, involving EMFs, is the exposure assessment, which is crucial to univocally link the appearance of the disease to the experienced exposure levels. In this case, the direct measure or numerical evaluation of the emitted EM field could be particularly hard and expensive, due to the elevated number of involved people and residential places (e.g., offices, houses, schools, or hospitals). So far, only a rough estimation of the dose has been possible, even based on people interview asking for the most common exposure sources present in their daily-life environment. Therefore, a more careful approach seems to be necessary in arranging new epidemiological campaigns. For instance, it could be useful to provide personal dosimeters, able to record in real time the effective EMFs levels, together with the time and the exact position of the exposure.

In this paper, we have revisited the experimental *in vitro* and *in vivo* studies, focused on the impact of the EMFs-driven oxidative pathway of the brain (Tables 1 and 2), as the high metabolic rate and the lipid rich composition of nervous system make this organ particularly sensitive to oxidative damage in both physiological processes and pathological conditions, such as neurodegeneration [46]. Indeed, the *in vivo* and *in vitro* experiments are able to provide more controlled, repeatable, and defined exposure conditions with respect to the epidemiological investigations, necessary to assess the dose-relationship studies and to set the hypotheses of related action mechanisms.

In this context, oxidative damage appears to be a master regulator of the biological response to EMFs in different cellular systems, together with alterations of blood parameters, changes in cytokine profiles, and effects on the immune system, although no clear understanding of the underlying mechanisms has been uniformly documented [15–19].

### 4.1. ELF-EMFs, Brain and Neurodegeneration

ELF stimulation, given as both short- (minimum 3 hours) and long-term (up to 10 months) exposure, seems almost univocally to be able to trigger oxidative stress (Table 1). In both animal brain and *in vitro* rat cortical neurons cultures, ELF-EMFs are associated to oxidative stress, that arises both from field interaction with chemical bonds of biomolecules, thus giving ROS a higher concentration and activity [51–55], and from disequilibrium in the enzyme-dependent scavenging ability [56–58]. In this ELF-ROS-brain context, only one paper by Kabuto et al. reported no ROS and no peroxidation effects following exposure of mice to ELF-EMFs [61], but description of the exposure and dosimetric details is poor.

A big controversy in disclosing ELF-EMFs effects in brain arises in the context of neurodegenerative diseases (Table 2). Epidemiological studies correlate occupational exposure to ELF-EMFs and AD and ALS pathogenesis, while poor epidemiological evidences have linked them to the onset and/or progression of both PD and HD [6, 9, 74, 94–97].

In AD pathogenesis, experimental findings propose melatonin biosynthesis, Ca^2+^ efflux in immune system and neurons, interference with the amyloidogenic process, as potential coeffectors of the ELF-mediated functions [78–81]. However, no univocal experimental findings by *in vitro* or *in vivo* studies have so far corroborated the hypothesis of the ELF-dependent oxidative stress as a key molecular regulator of the AD development. 

In the ALS context, an attempt to assess a functional correlation between ELF and neurodisease has been carried out exclusively by De Gannes et al. [88], in an oxidative stress-prone experimental model of Tg (SOD1^G93A^) mice, at the moment the most accurate animal model for studying this pathology. By precisely monitoring body weight, motor function, and life span, authors did not report any significant redox-related change in Tg-exposed mice, although exposure was carried out over a 7 weeks period. Whether a longer treatment or exposure of younger animals would affect the outcome is unknown, and definitely requires further investigations, also in additional experimental animal models that do not exclusively represent the ALS familial (mutated SOD) form.

In the research field of PD, although not described in this paper, it is worth mentioning the presence of different studies in favor of possible therapeutic potentials of the so-called transcranial magnetic field stimulation (TMFS) in the frequency range of the ELF [116]. TMFS is a relatively innovative technique applied to investigate corticospinal physiology and other properties of the primary motor cortex, such as excitability [117, 118]. Even though no involvement of oxidative stress has been so far reported, some records claim that TMFS is able to relief patients from most parkinsonian symptoms, driving amelioration of the reaction and movement time, of the performance on the grooved pegboard test in patients whose dominant motor hand area was stimulated by a focal coil during testing [117]. These data may suggest a protective function of ELF, but TMFS is based on single- or paired-pulsed signal that cannot be properly considered as an ELF-EMF. Besides, there are no experimental data supporting clinical observations, and further animal studies may shed some light on the mechanisms involved and perhaps provide a stronger rationale for improvement of patients afflicted with PD treated with TMFS therapy. 

Convincing experimental evidences, in support of a potential neuroprotective effect of ELF exposure, have been produced exclusively in HD animal models. Exposure to ELF-EMFs (administered as both short term treatment, for 8 days, and for long term exposure of 21 days) has been indeed reported to significantly prevent and reverse the oxidant effect induced by the neurotoxin 3NP [89–91]. It needs to be highlighted that all these set of experimental findings, carried out in the 3NP-treated Wistar rats, origins from the same research group. Besides, in the experimental procedures, the authors refer improperly to a transcranial magnetic stimulation (TMS) exposure, while TMS signals have completely different characteristics from those applied by Tùnez' group [89–91]. What they used is a simple sinusoidal ELF signal, while real TMS stimulation consists in a mophasic or bipahsic pulse (e.g., a dumped cosine) provided to the biological sample in multiple trains at a repetition frequency of tens of Hz, as well described by Peterchev et al. [119]. 

Hence, it is now well accepted that ELF-EMFs influence the *in vitro* behavior of numerous cell types, and that these changes trigger diverse effects which may have positive or negative outcomes, depending on the cell type [120–122]. This phenomenon could partially explain the opposite results obtained in different *in vitro* studies, but does not give rise to any explanation for opposite findings in animal models upon ELF exposure in brain. It has been postulated that ELF stimulation can affect physiology of neurons by inducing oxidative damage, lipid peroxidation, and neurotransmitter release. These data might suggest a possible prodegenerative effect of ELF, as the oxidative stress is clearly a hallmark of neurodegeneration. Unexpectedly, a completely different response is elicited if ELF stimulation is administered to neurons that are still compromised by an early event of neurodegeneration, and/or if applied over a long period. Like in other diseases, such as cancer, it is often a matter of balance between opposite stimuli, and a matter of when the external stress factor is hitting the cell, whether in early or late degenerative step. 

In addition, it is worth to notice that an appropriate description of the ELF-EMFs homogeneity within the used exposure device, as well as temperature control, is lacking in the majority of the exposure configurations and protocols reviewed, in contrast to the requirements for controlled and high quality experiments in bioelectromagnetic reported by Kuster for low-frequency fields [123]. Moreover, at these frequencies, sham control is a crucial issue that needs to be carefully implemented. Normally, the exposure systems are turned off to obtain such a condition, while a more appropriate sham exposure should be represented by coil systems using separated strand cables wrapped in parallel to enable the currents flowing also in antiparallel (sham) directions. Only in this way, it is possible to reproduce exactly the same environmental conditions of the exposed case in term of vibrations and temperature variations.

### 4.2. HF-EMFs, Brain, and Neurodegeneration

The experimental evidences linking the field exposure to the oxidative stress in brain and neurodegeneration are controversial also in the context of the HF-EMFs. The influence of RF on biological systems, in particular the presence of biological effects on and risk to humans, has been a subject of intense debate for several decades. Recently, this debate intensified due to new applications of RF-EMFs in cordless stationary phones, wireless computer communication, and, most importantly, due to the exploding use of mobile phones. Since the quantum energy of RF-EMFs is extremely low compared to ionizing radiation, it is plausible that no conclusive and reproducible genotoxic effects, such as increased DNA damage or increased mutation rates, will be observed in response to RF-EMFs. Since interactions between RF-EMFs and certain molecules in biological systems form the basis for possible RF-EMFs-induced changes in these systems, it has been assumed that only the absorbed radiation from RF-EMFs can have effects in biological systems. Hence, the specific absorption rate should be a key measure for the induction of biological effects. Most of the RF-EMFs radiation absorbed is converted into increased thermal energy of the system [35], which is responsible for most effects observed in biological systems. Nevertheless, it is now well accepted that also low-level EMF exposure, which does not induce thermal effect, could carry a biological response. So, a major experimental problem is the definition of non-thermal effects and how to distinguish them from direct and indirect thermal effects [36–38]. 

One of the hypothesized targets for nonthermal effect of RF-EMFs is the oxidative stress, although experimental *in vitro* and *in vivo* findings in brain are contradictory, ranging from prooxidant ability of GSM exposure observed in primary cortical neurons cultures and in animal model [63–66], to no-effect reported in SH-SY5Y (human neuroblastoma) and L929 (mouse fibroblasts) cell lines and in mice brain and sera [37, 67] (Table 2). This overall contradiction in neuronal parameters in response to RF definitely reflects the uncertainty in identifying the molecular effects driven by GSM, and in distinguishing between thermal and nonthermal ones.

The scenario in neurodegeneration response to RF stimulation has been recently revisited following data from Arendash and colleagues [82–84] (Table 2). They demonstrated for the first time that long term RF stimulation provides cognitive benefits to AD animals, disclosing a potential noninvasive, nonpharmacological therapeutic strategy against Alzheimer's. In accordance to a potential RF-driven neuroprotective effect (although exclusively supported by *in vitro* evidences), low SAR GSM-900 MHz exposure has been reported to downregulate the *α*-synuclein in a highly (80%) neuron-enriched mixed cortical cell culture from brains of rat embryos [87], suggesting a hypothetic beneficial effect of these frequencies also in PD model (Table 2).

In the RF-induced neuroprotection of AD models, authors demonstrate that all the cognitive benefits occur without induction of brain hyperthermia and without increase in brain oxidative stress [82, 84]. Surprisingly, experimental data from the same group attributed the long term-RF-dependent effects to the enhancement of brain mitochondrial function of AD transgenic (Tg) animals [85], in terms of reduced mitochondrial ROS generation and enhanced mitochondrial membrane potential, in both cerebral cortex and hippocampus of AD Tg mice. These findings are in contrast to what is stated in the other two publications (where they reported no change in the indices of brain oxidative stress), and leaves whether GSM functions involve oxidative stress or not.

Moreover, major concerns remain on the exposure system employed by Arendash' group and on the dosimetric assessment performed to define the mentioned SAR levels. First, the provided SAR calculation does not specify if it is referred to the internal field levels (within mouse) or to the external ones. In this last case, the reported SAR values have no sense, as SAR is defined as the absorbed dose in the unitary mass of the biological target (a mouse in this case) within a certain time interval. Besides, it is not accurate to perform a SAR calculation that does not take into consideration the different conductivities and densities of the animal tissues. In this case, a sort of average value, for both conductivity and density, has been used, rendering the SAR estimation within the biological target extremely approximate. Also, no information about field homogeneity inside the exposure target is provided. This observation leads to the conclusion that the performed evaluation cannot be considered as a satisfactory dosimetry for the target. The methodology employed for field measurements should be clearly stated, and further EM simulations required to confirm the experimental SAR values, as well noted in Kuster and Schönborn 2000 [123]. Without a rigorous dosimetry (local and mean SAR values obtained both experimentally and numerically, plus evaluation of the SAR homogeneity), the real delivered dose within mice remains unknown, consequently making unreliable and completely nonreplicable the obtained results.

On the basis of both Arendash' results, and other evidences that TTR can bind A*β*, and thus protect against its deposition [124], Söderqvist et al. evaluated TTR levels in people exposed to GSM [86]. He describes an increase of TTR after GSM signal exposure, and argues that the hypothetic RF effect on AD could be TTR-mediated. A number of concerns arise with respect to the methodology chosen for the analysis. For cross-sectional study, people were asked to answer a postal questionnaire about use of mobile phones and cordless phones. This is a widely adopted solution in epidemiological studies on EM fields, leading to a series of mistakes related to the assessment of the exposure. Indeed, the information provided cannot be always complete and accurate. For provocational study, the EMFs exposure was performed at 890 MHz GSM signal for 30 min. A homogenous specific absorption rate (SAR 1 g) of 1.0 Wkg^−1^ to the temporal area was applied. However, authors do not specify how this SAR value was assessed. May be, numerical simulations were performed. In addition, the system used to deliver the EM fields close to human head is not described. Hence, it is difficult to effectively evaluate the dose and consequently to replicate the study.

Therefore, depending on the dose, the frequency, the exposure period, EMFs are reported to be either harmful or protective in neuronal response, suggesting even a possible application in medical therapy. Hence, so far no univocal interpretation of the EMFs effects in brain and neurodegeneration can be proposed, as epidemiological studies are difficult to be carried out, *in vitro* and *in vivo* models are heterogeneous, and laboratory exposure set-ups often present limitations without a proper dosimetry. The experimental conditions in the EMFs experiments, such as the induced field within the biological target, its frequency, as well as the impulse shape, and time of exposure, may affect biological response. Conflicting biological data might be thus attributable to differences in the frequency and intensity of the field, exposure time, heat generation, cell penetration, and experimental model considered. When RF exposure effects are investigated, it has to be considered that the biological samples modify the systems performances; hence, the features of the exposure devices have to be rigorously evaluated during their design steps and final characterization. As a consequence, the dosimetric assessment within the biological targets is of primary importance for well-controlled experiments [123]. In particular, Kuster and Schönborn [123] established that the required SAR homogeneity for high-quality investigations has to be of the order of 70%. This quantity should be assessed by using both experimental methodologies (e.g., EMF and SAR measurements) and numerical EM simulations, capable of describing precisely the biological target geometry and its electric properties, as well highlighted in different papers [125–128].

We would also like to stress that in a number of *in vitro* and *in vivo *studies performed at RF and MW frequencies, unacceptable exposure conditions for cell phones, in direct contact to the cell cultures or animals, have been employed [37, 82, 85, 129, 130]. This exposure conditions do not guarantee any control of the emitted power and thus of the SAR induced within the samples. 

In the light of results reviewed here, we can conclude that there are no incontrovertible evidences of the role of EMFs in oxidative stress modulation. Hence, it is mandatory to proceed with intense research on this issue, paying particular attention to the choice of the appropriate biological model and well-controlled experimental conditions.

## Figures and Tables

**Table 1:** EMFs exposure and oxidative stress in brain.

Type of EMFs	EMFs exposure details	EMFs effect	Experimental model	Reference
	50 Hz, 0.1–1.0 mT, 7 days	Prooxidant	Cortical neurons (Spraque-Dawley rat embryo)	Di Loreto et al. [51]
	60 Hz, 2.3 mT, 3 hours	Prooxidant	ICR Mouse cerebellum	Chu et al. [52]
	40 Hz, 7 mT, 30 min/day for 10 days	Prooxidant	Spraque-Dawley rat brain	Ciejka et al. [53]
	50 Hz, 0.5 mT, 7 days	Prooxidant	Wistar rat brain	Jelenković et al. [54]
ELF	50 Hz, 50 mG, for 5 min/day for 6 months	Prooxidant	Spraque-Dawley rat brain	Bediz et al. [55]
	60 Hz, 12 G, 3 hours	Prooxidant	Balb/c mice brain	Lee et al. [56]
	50 Hz, 100 and 500 *μ*T, 2 hours/day for 10 months	Prooxidant	Spraque-Dawley rat brain	Akdag et al. [57]
	60 Hz, 2.4 mT, 2 hours	Prooxidant	Wistar rat brain	Martínez-Sámano et al. [58]
	50 Hz, 0.1–1.0 mT, 10 days	Prooxidant	Spraque-Dawley rat brain	Falone et al. [60]
	60 Hz, 0.2–1.2 mT	No oxidative effect	ICR mouse brain	Kabuto et al. [61]

	900 MHz, SAR of 2 Wkg^−1^, 7 days	Prooxidant	Wistar rat brain	Ilhan et al. [63]
	890–915 MHz, SAR 0.95 Wkg^−1^, for 12 h/day for 30 days	Prooxidant	Guinea pig brain	Meral et al. [64]
RF	900 MHz, SAR of 1.5 Wkg^−1^, and 6 Wkg^−1^, 7 days	Prooxidant	Spraque-Dawley rat brain	Ammari et al. [65]
	1800 MHz, SAR of 2 Wkg^−1^, 24 hrs	Prooxidant	Primary cortical neuronal cultures (new-born SD rats)	Xu et al. [66]
	900 MHz, 0.02 mWcm^−2^, 30 min/day for 7 days	No oxidative effect	New Zealand rabbit brain	Irmak et al. [37]
	872 MHz, SAR of 5 Wkg^−1^, 1 hour and 24 hours	No oxidative effect	SHSY5Y and L929 cells	Höytö et al. [67]

**Table 2:** EMFs effects on oxidative stress and neurodegeneration: *in vitro *and *in vivo *experimental models.

Pathology	EMFs exposure details	EMFs effect	Experimental model	Reference
	RF: 918 MHz, SAR 0.25 WKg^−1^ 7–9 months	Cognitive benefits No brain oxidative stress	Tg(A*β*PPsw ) and non-Tg mice	Arendash et al. [82]
AD	RF: 918 MHz, SAR 0.25 and 1.05 WKg^−1^ 1 hour/day for 1 month	Cognitive benefits Decreased mitochondria oxidative stress in Tg mice	Tg(A*β*PPsw + PS1) and non-Tg mice	Dragicevic et al. [85]
	RF: 918 MHz, SAR 0.25 and 1.05 WKg^−1^ 2 hour/day for 2 months	Cognitive benefits Decreased brain A*β* deposition, No brain oxidative stress	Aged Tg(A*β*PPsw + PS1) and non-Tg mice	Arendash et al. [84]

PD	RF: 900 MHz, SAR 0.25 WKg^−1^ 24 hours	Down-regulation of *α*-synuclein No oxidative stress	Neuron-enriched mixed cortical cell culture from brains of rat embryos (Wistar rats)	Terro et al. [87]

ALS	ELF: 50 Hz, at 100 and 1000 T 2 hours/day, 5 days/week for 7 weeks	No effect	Tg (SOD1^G93A^) and non-Tg mice	Poulletier De Gannes et al. [88]

	ELF: 60 Hz, 0.7 mT, 2 hours in the morning + 2 hours in the afternoon, for 8 days	Neuroprotective Decreased oxidative stress	3NP-treated Wistar rats	Túnez et al. [89]
HD	ELF: 60 Hz, 0.7 mT, 2 hours in the morning + 2 hours in the afternoon, for 8 days	Neuroprotective Decreased GSH, GSH-Px, CAT levels	3NP-treated Wistar rats	Túnez et al. [90]
	ELF: 60 Hz, 0.7 mT, 21 days	Neuroprotective Decreased oxidative stress	3NP-treated Wistar rats	Tasset et al. [91]

## Abbreviations

A*β*: Amyloid beta
AD: Alzheimer's disease
ADA: Adenosinedeaminase
ALS: Amyotrophic lateral sclerosis
APP: Amyloid precursor protein
CAT: Catalase
DC: Direct current
E-field: Electric field
EHC: Environmental health criteria
ELF: Extremely low frequency
EM: Electromagnetic
EMF: Electromagnetic field
GSH: Glutathione
GSH-Px: Glutathione peroxidase
GSM: Global system for mobile communications
HD: Huntington's Disease
HF: High frequency
H-field: magnetic field
IARC: International Agency for Research on Cancer
MDA: Malondialdehyde
MPO: Myeloperoxidase
MRI: Magnetic resonance imaging
MW: Microwave
3NP: 3-Nitropropionic
NO: Nitric oxide
PD: Parkinson's disease
PS1: Presenilin
RF: Radio frequency
ROS: Reactive Oxygen species
SAR: Specific absorption rate
SDH: Succinate dehydrogenase
SOD: Superoxide dismutase
Tg: Transgenic
TMS: Transcranial magnetic stimulation
TMFS: Transcranial magnetic field stimulation
TTR: Transthyretin
WHO: World Health Organization
XO: Xantine oxidase.
